# Stereoscopic Rendering *via* Goggles Elicits Higher Functional Connectivity During Virtual Reality Gaming

**DOI:** 10.3389/fnhum.2019.00365

**Published:** 2019-10-25

**Authors:** Caroline Garcia Forlim, Lukas Bittner, Fariba Mostajeran, Frank Steinicke, Jürgen Gallinat, Simone Kühn

**Affiliations:** ^1^Department of Psychiatry and Psychotherapy, University Medical Center Hamburg-Eppendorf (UKE), Hamburg, Germany; ^2^Department of Human-Computer-Interaction, University of Hamburg, Hamburg, Germany; ^3^Max Planck Institute for Human Development, Lise-Meitner Group for Environmental Neuroscience, Berlin, Germany

**Keywords:** virtual reality, stereoscopic and monoscopic goggles, fMRI, seed-based functional connectivity, fractional amplitude of low-frequency fluctuations, resting-state networks, ICA, graph analysis

## Abstract

Virtual reality (VR) simulates real-world scenarios by creating a sense of presence in its users. Such immersive scenarios lead to behavior that is more similar to that displayed in real world settings, which may facilitate the transfer of knowledge and skills acquired in VR to similar real world situations. VR has already been used in education, psychotherapy, rehabilitation and it comes as an appealing choice for training intervention purposes. The aim of the present study was to investigate to what extent VR technology for games presented *via* goggles can be used in a magnetic resonance imaging scanner (MRI), addressing the question of whether brain connectivity differs between VR stimulation *via* goggles and a presentation from a screen *via* mirror projection. Moreover, we wanted to investigate whether stereoscopic goggle stimulation, where both eyes receive different visual input, would elicit stronger brain connectivity than a stimulation in which both eyes receive the same visual input (monoscopic). To our knowledge, there is no previous research using games and functional connectivity (FC) in MRI to address this question. Multiple analyses approaches were taken so that different aspects of brain connectivity could be covered: fractional low-frequency fluctuation, independent component analysis (ICA), seed-based FC (SeedFC) and graph analysis. In goggle presentation (mono and stereoscopic) as contrasted to screen, we found differences in brain activation in left cerebellum and postcentral gyrus as well as differences in connectivity in the visual cortex and frontal inferior cortex [when focusing on the visual and default mode network (DMN)]. When considering connectivity in specific areas of interest, we found higher connectivity between bilateral superior frontal cortex and the temporal lobe, as well as bilateral inferior parietal cortex with right calcarine and right lingual cortex. Furthermore, we found superior frontal cortex and insula/putamen to be more strongly connected in goggle stereoscopic vs. goggle monoscopic, in line with our hypothesis. We assume that the condition that elicits higher brain connectivity values should be most suited for long-term brain training interventions given that, extended training under these conditions could permanently improve brain connectivity on a functional as well as on a structural level.

## Introduction

Virtual reality (VR) is used in various contexts such as entertainment, education, psychotherapy, rehabilitation and other conditions. In these computer-generated environments, the user can perceive, feel and interact in a manner that is similar to a physical place, which is usually achieved by a combination of multiple sensory channels, such as sight, sound and touch. An essential feature of VR is that it creates a sense of presence in its users, meaning a sense of being in a virtual environment that is more engaging than the surrounding world (Slater and Wilbur, 1997), which in turn leads to behavior that is more similar to the behavior displayed in real world settings. Importantly this feeling of presence may facilitate transfer of knowledge and skills acquired in VR to similar real-world situations, which would make VR an ideal choice for training intervention purposes.

In preparation for such training studies using games and to test to what extent VR technology for games presented *via* goggles can be used in a magnetic resonance imaging scanner (MRI) we conducted the present study. We set out to investigate whether VR visual stimulation using MRI compatible goggles with 3D stereoscopic stimulation (in which the image is rendered separately for each eye creating the illusion of depth and 3D effect) differs in terms of brain connectivity from more commonly applied presentation forms using goggles with 2D monoscopic presentation (in which both eyes receive the same visual input) and a conventional screen back-projection *via* a mirror. Our hypothesis is that the condition that elicits higher brain connectivity values should be most suited for long-term brain training interventions given that, extended training under these conditions could permanently improve brain connectivity. To study potential brain connectivity differences elicited by 3D stereoscopic, 2D monoscopic and screen stimulations, we chose multiple methods, each of them being able to reveal different aspects of brain connectivity: independent component analysis (ICA) a data-driven technique to extract whole-brain networks, seed-based functional connectivity (SeedFC) that calculates the brain network related to specific regions of interest (ROIs) and graph analysis that characterizes the topology of the brain networks. Additionally, we tested brain activation in the domain of low-frequency fluctuation of the blood-oxygen-level dependent (BOLD) signal using fractional amplitude of low-frequency fluctuation (fALFF). To our knowledge, there is no previous research using games and FC in MRI to address this question. Most of the previous studies either used different stimulus material to investigate different degrees of spatial presence (Lee et al., 2005; Baumgartner et al., 2006, 2008; Havranek et al., 2012; Dores et al., 2013) and/or used electroencephalography (EEG; Baumgartner et al., 2006; Havranek et al., 2012; Kober et al., 2012; Slobounov et al., 2015; Dan and Reiner, 2017). Gaebler et al. (2014) employed the same movie as stimulus delivered in 3D stereoscopic and monoscopic 2D and evaluated the subject experience and intersubject correlation of brain activity finding higher immersion and a more realistic stimulus was associated with higher intersubject correlations. Nevertheless, the connectivity between brain areas was not investigated.

Most interesting for the present endeavor and the question whether VR elicits higher brain connectivity, is an EEG study that compared brain signals during navigation either on a desktop PC (2D) or on a large wall projection in 3D (Kober et al., 2012). The 3D condition was accompanied by higher cortical parietal activation in the alpha band, whereas the 2D condition was accompanied by stronger FC between frontal and parietal brain regions, indicating enhanced communication. In two additional EEG studies, in which different modes of presentation have been compared, but in which brain activity instead of connectivity was the focus of investigation, contradictory evidence has been gathered. Likewise in a navigation task comparing a condition in which participants wore 3D glasses and watched a screen vs. a 2D screen condition, higher theta power in frontal midline structures was observed in the 3D VR condition (Slobounov et al., 2015). In contrast, a study investigating paper folding (origami) learning with 3D glasses vs. with a 2D film showed that the 2D condition displays a higher so-called cognitive load index computed as the ratio of the average power of frontal theta and parietal alpha. A last study focused on intra-hippocampal EEG recordings comparing real-world navigation vs. VR navigation and demonstrated that oscillations typically occurs at a lower frequency in virtual as compared to real-world navigation (Bohbot et al., 2017).

Brain regions that have repeatedly received attention in the endeavor to explain differences between VR-related presence experiences in comparison to 2D or less immersive environments are the prefrontal cortex (PFC), the parietal cortex as well as the hippocampus (Baumgartner et al., 2006, 2008; Beeli et al., 2008; Kober et al., 2012; Bohbot et al., 2017; Dan and Reiner, 2017). For this reason, we used whole-brain connectivity analysis approaches as well as ROI-based approaches focusing on the effects of the type of the display (conventional 2D screen, MRI goggles with monoscopic view effect, MRI goggles with stereoscopic view effect) which capture two different degrees of immersion in a technical (Slater and Wilbur, 1997) and subjective (Gaebler et al., 2014) point of view.

## Materials and Methods

### Participants

Twenty-six healthy participants were recruited from a local participant pool. After complete description of the study, the participants’ informed written consent was obtained. Exclusion criteria for all participants were abnormalities in MRI, relevant general medical disorders and neurological diseases. Additional exclusion criteria were movement above the threshold of 0.5 (Power et al., 2012) during the scanning section and completion of all three conditions. After the exclusion criteria were applied, the number of subjects dropped to 23 (mean age = 26.5, SD = 4.8 years, female:male = 11:12). The protocol was approved by the local psychological ethics committee of the University Medical Center Hamburg-Eppendorf, Germany. All subjects gave written informed consent in accordance with the Declaration of Helsinki.

### Game Description and Procedure

**Figure 1:**
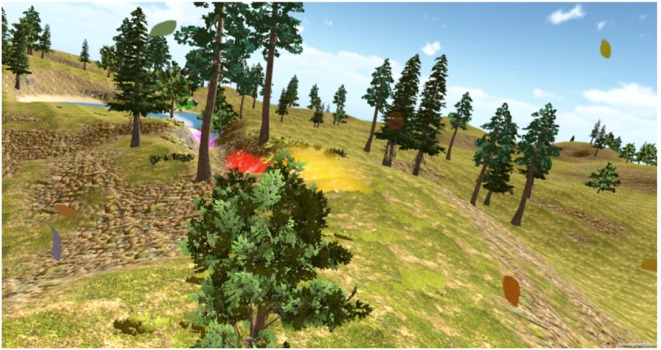
A screenshot of the player’s view when flying in the virtual landscape. The colorful halos indicate the positions of the next flowers to be “pollinated.”

While situated in the scanner, participants were exposed to a game (FlowVR) that was inspired by the game Flower by Thatgame Company^1^ and programmed in Unity. The player flies through a nature scene with the goal of making the landscape blossom (Figure 1). This can be achieved by the player flying close to flowers, which are surrounded by a colored halo, to virtually pollinate them so that more flowers grow in the surrounding area. Visual and auditory elements associated with positive affect have been implemented. The task has been designed with the goal to reduce negative affect and initiate the experience of flow (Csikszentmihalyi, 2014).

Before entering the scanner, the participants were given information about the gameplay. The participants did not practice outside the scanner. They were asked to imagine to be a bee, whose goal is to make the landscape flourish by flying above it and touching flowers or trees which are surrounded by a bright halo. That would lead to pollen popping out of the flowers and new flowers, bushes or trees growing all around. They were told to follow the path of the flowers and collect as many flowers as possible, however, they were likewise instructed to not mind when missing out on a flower. The number of pollinated flowers was not counted and no score was kept. For this reason, no behavioral measures of the game were reported. Participants used an MR compatible button box with four buttons in a row from Nordic Neuro Lab to navigate in the game. The user had to hold the controller with both hands and use the two left buttons for flying upwards and downwards and the other two for flying to the left and right. Independently of how many flowers were pollinated, the speed of the flight was kept constant and each run lasted about 5 min.

Participants underwent three conditions, one in which the game was projected from a screen *via* mirror projection, one in which MRI compatible goggles were used either with a 3D stereoscopic stimulation (in which the image is rendered separately for each eye creating the illusion of depth and 3D effect) and a 2D monoscopic presentation (in which both eyes received the same visual input). MRI compatible goggles used were the VisuaStim digital^2^, with a resolution of 800 × 600 (SVGA) and field of view (FoV) of 30° horizontal and 24° vertical. The order of the conditions was randomly assigned to the participants. Due to subject exclusion the order of condition, monoscopic-stereoscopic—screen were performed for three subjects instead of four (23 subjects distributed among six orders of condition).

### Scanning Procedure

Structural images were collected on a Siemens Prisma 3T scanner (Erlangen, Germany) and a standard 32-channel head coil was used. The structural images were obtained using a three-dimensional T1-weighted magnetization prepared gradient-echo sequence (MPRAGE; repetition time = 2,500 ms; echo time = 2.12 ms; TI = 1,100 ms, acquisition matrix = 240 × 241 × 194, flip angle = 9°; 0.8 × 0.8 × 0.94 mm voxel size). Resting-state data were acquired after the T1 image. We acquired whole-brain functional images while participants were asked to keep their eyes closed and relax for 5 min. We used a T2*-weighted echo-planar imaging (EPI) sequence (repetition time = 2,000 ms, echo time = 30 ms, image matrix = 64 × 64, field of view = 216 mm, flip angle = 80°, slice thickness = 3.0 mm, distance factor = 20%, voxel size = 3 × 3 × 3 mm^2^, 36 axial slices, using GRAPPA). Images were aligned to the anterior-posterior commissure line.

### Functional MRI Data Analysis

#### Preprocessing

To ensure for steady-state longitudinal magnetization, the first 10 images were discarded. Slice timing and realignment were performed in the remaining images. The individual anatomical images T1 were coregistered to functional images and segmented into white matter, gray matter, and cerebrospinal fluid. Data were then spatially normalized to Montreal Neurological Institute (MNI) space and spatially smoothed with an 8-mm FWHM to improve signal-to-noise ratio. Signal from white matter, cerebrospinal fluid and movement were regressed. To reduce physiological high-frequency respiratory and cardiac noise and low-frequency drift data was filtered in the bandwidth of (0.01–0.08 Hz) and, finally, detrended. All steps of data preprocessing were done using SPM12 (Wellcome Department of Cognitive Neurology) except filtering that was applied using REST toolbox (Song et al., 2011). In addition, to control for motion, the voxel-specific mean framewise displacement (FD) was calculated according to Power et al. (2012). We excluded from the analyses participants who had an FD above the recommended threshold of 0.5.

#### Fractional Amplitude of Low-Frequency Fluctuation (fALFF)

fALFF is not a measure of connectivity between areas, but rather it accounts, voxel-wisely, for changes in the amplitude of low frequency spontaneous fluctuations in the BOLD imaging signal in the whole brain. fALFF represents the relative contribution of low frequency oscillations to the total detectable frequency range and is calculated taking the power within a frequency range and dividing it by the total power in the whole detectable frequency range (Zou et al., 2008). For that, first the voxel time series are transformed into the power domain, then the amplitudes within a specific low-frequency bandwidth are summed. Finally, this value is divided by the sum of the amplitudes in the entire detectable frequency range. To create standardized maps, each subject maps is transformed into Z-scores. We calculated the fALFF using REST toolbox (Song et al., 2011). Subject-specific fALFF maps were taken to the second level analysis in SPM12.

#### Independent Component Analysis (ICA)

ICA a data-driven technique to extract whole-brain networks. We examined the resting-state networks given by the spatial grouping of voxels with temporally coherent activity calculated in a data-driven fashion using ICA. ICA decomposes blindly, in multiple independent components, the brain activity. Each component is a spatial grouping of voxels with temporally coherent activity (connectivity) and according to this spatial grouping of voxels, the components are associated with sources that can be either related to brain activity or to noise such as movement, blinking, breathing, and heartbeat. The main brain activity-related sources resemble discrete cortical functional networks and are named resting state networks (RSN). The RSN comprise the default mode network (DMN), basal ganglia, auditory, visuospatial, sensory-motor, salience, executive control, language and visual networks. Here, ICA was performed in GIFT software^3^ using Infomax algorithm. The number of spatially independent resting-state networks (N) was estimated by the GIFT software (*N* = 26). The identification of the networks was done automatically using predefined GIFT templates and later the resting-state networks of interested, the DMN and visual networks, were chosen by two specialists (CGF and SK). For every resting-network of interest, subject-specific spatial connectivity maps were taken to the second level analysis in SPM12.

#### Seed-Based Functional Connectivity (SeedFC)

FC is one of the most popular methods to infer connectivity in neuroimaging. When FC is calculated by means of the temporal correlations (Pearson’s correlation) between a ROI to the other voxels in the whole brain, it is known as seed-based FC (SeedFC). In this way, SeedFC calculates the brain network related to specific ROIs. We investigated the seed-based connectivity maps, using as seed the brain ROIs in VR, namely, bilateral superior and middle frontal cortex, bilateral hippocampus, bilateral superior and inferior parietal cortex, areas shown in previous works that may explain differences between VR-related presence experiences and less immersive environments (Baumgartner et al., 2006, 2008; Beeli et al., 2008; Kober et al., 2012; Bohbot et al., 2017; Dan and Reiner, 2017). The seed areas were defined using the anatomical automatic labeling atlas (AAL). Seed-based connectivity maps were obtained by correlating the seed time series with all voxels in the brain. For that, first we extracted the time series of all voxels within the corresponding ROI delimitated by the AAL atlas and then we took the average. Next, we calculated the Pearson’s correlation coefficient between the seed time series and all other voxels in the brain. Finally, Fischer transformation was applied to the individual FC maps obtaining Z scores to improve normality. The individual seedFC maps were calculated in MATLAB R2012b^4^. The Z score maps were taken to the second level in SPM 12 (Statistical Parametric Mapping package; Wellcome Department for Imaging Neuroscience, London, United Kingdom^5^.

#### Graph Analysis

To examine differences in the topology of the brain networks we performed graph analysis (Bullmore and Sporns, 2009). The first step was to construct the FC matrices, where nodes and links should be defined. Nodes were brain regions created based on the AAL116 atlas (Tzourio-Mazoyer et al., 2002) and the links were the connectivity strength between nodes calculated using Pearson’s correlation coefficient. The node-averaged time series used to infer the connectivity strength were extracted for each subject using the REST toolbox (Song et al., 2011). To avoid false-positive links, connectivity values that were not statistically significant (*p*-value ≥ 0.05) were excluded. Once the functional matrices are built, graph analysis can be applied in order to characterize their topology. At this stage, thresholding was applied, namely density threshold ranging from 0.1 to 0.8 in steps of 0.1. Thresholding means that only links with the highest connectivity strengths are kept until the desired density is reached, e.g., a threshold of 0.1 means 10% of the links with the highest connectivity were kept and the remaining ones were set to 0. Graph analysis was then applied to these thresholded matrices using the Brain Connectivity toolbox (brain-connectivity-toolbox.net). The main graph measures were chosen: betweenness centrality that measures the fraction of all shortest paths that pass through an individual node; characteristic path length which accounts for the average shortest path between all pairs of nodes; efficiency which is the average inverse shortest paths and transitivity that measures the relative number of triangles in the graph, compared to total number of connected triples of nodes. For a complete description of the graph measures please refer to Bullmore and Sporns (2009) and Rubinov and Sporns (2010).

### Statistics

We were interested in two particular contrasts: (1) VR visual stimulation using MRI compatible goggles with 3D stereoscopic stimulation and 2D monoscopic presentation vs. screen *via* mirror projection, to investigate for brain differences during goggles and screen; and (2) 3D stereoscopic stimulation, in which the image is rendered separately for each eye creating the illusion of depth and 3D effect vs. 2D monoscopic presentation, most commonly applied presentation in which both eyes receive the same visual input, so that we could investigate the effect of stereoscopic view in the brain.

The resulting individual maps of each analysis were taken to the second level analysis in SPM12 (Statistical Parametric Mapping package; Wellcome Department for Imaging Neuroscience, London, United Kingdom^6^) with mean FD as covariate. Using a family wise error (FWE) threshold on the cluster level of *p* < 0.05 (cluster extent = 50) we ran the two contrasts in both the positive and the negative direction.

For the graph analysis, repeated measures one-way ANOVA was used. FDR was used for multiple comparison correction.

## Results

Since we were mostly interested in the direction of potential brain connectivity differences between conditions, we employed multiple different analysis pipelines. We employed four methods that focus on different aspects of the brain signal, the intrinsic frequency fluctuation and the connectivity given by the spatial grouping of voxels with temporally coherent activity and by the temporal correlations between areas, respectively, fALFF that measures the amplitude of the low frequency fluctuation of the BOLD signal, ICA that uncovers the resting-state brain networks, the seed-based FC that calculates the brain network related to specific ROIs and graph analysis that characterizes the topology of the brain networks. Results are summarized in Table 1.

**Table 1:** Group differences in fractional amplitude of low-frequency fluctuation (fALFF), independent component analysis (ICA) and seed-based functional connectivity (Seed-FC) analyses.

Analysis	Contrast	Labels	MNI coordinates	*T*	Cluster size (in voxels)	*P* (cluster level FWE corrected)
fALFF	goggles > screen	Left cerebellum VI	−20 −62 −26	4.32	92	0.007
		Left postcentral gyrus	−42 −40 60	4.05	93	0.007
	goggles < screen	Right frontal superior orbital	18 56 −4	6.13	72	0.006
ICA Primary visual	goggles < screen	Bilateral cuneus	6 −80 30	6.25	365	< <0.001
		Left middle occipital	−18 −98 −2	4.94	108	0.025
	goggles > screen	Left calcarine	−8 −94 10	4.6	152	0.018
ICA Higher visual	goggles < screen	Bilateral cuneus	0 −78 24	4.7	232	0.001	
	goggles > screen	Left lingual	−4 −64 4	6.2	257	< <0.001
ICA	goggles > screen	Bilateral lingual	−2 −66 0	5.1	192	0.003
DMN		Inferior frontal gyrus/precentral gyrus	−34 0 28	5.0	324	< <0.001
SeedFC	goggles > screen	Left superior temporal pole	−48 16 −12	4.7	332	< <0.001
Bilateral superior frontal	stereoscopic > monoscopic	Left insula/putamen	−34 10 10	5.4	237	< <0.001
SeedFC Bilateral inferior parietal	goggles > screen	Right calcarine	18 −98 4	4.5	182	0.002
Bilateral inferior parietal		Right lingual	26 −88 −6			
		Right calcarine	20 −88 2			

### fALFF

**Figure 2:**
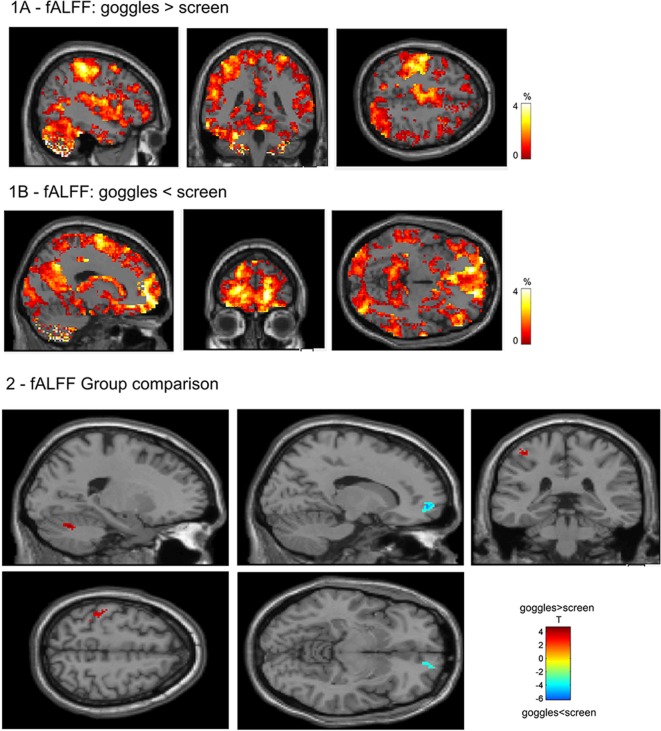
1—Difference of the mean fALFF maps, 1A goggles > screen and 1B goggles < screen. 2—Group differences in fALFF. Higher fALFF (in red) in left cerebellum and left postcentral gyrus in the goggles (monoscopic + stereoscopic) condition compared with the screen condition. In the reverse contrast (in blue) there was higher fALFF in right superior orbital frontal cortex.

In fALFF, we found significantly higher fALFF (Figure 2) in left cerebellum (VI, −20, −62, −26), left postcentral gyrus (−42, −40 60) in the goggles (monoscopic + stereoscopic) condition compared with the screen condition. In the reverse contrast, we observed higher fALFF in right superior orbital frontal cortex (−18, 56, −4; all results FWE corrected *p* < 0.05).

### ICA

**Figure 3:**
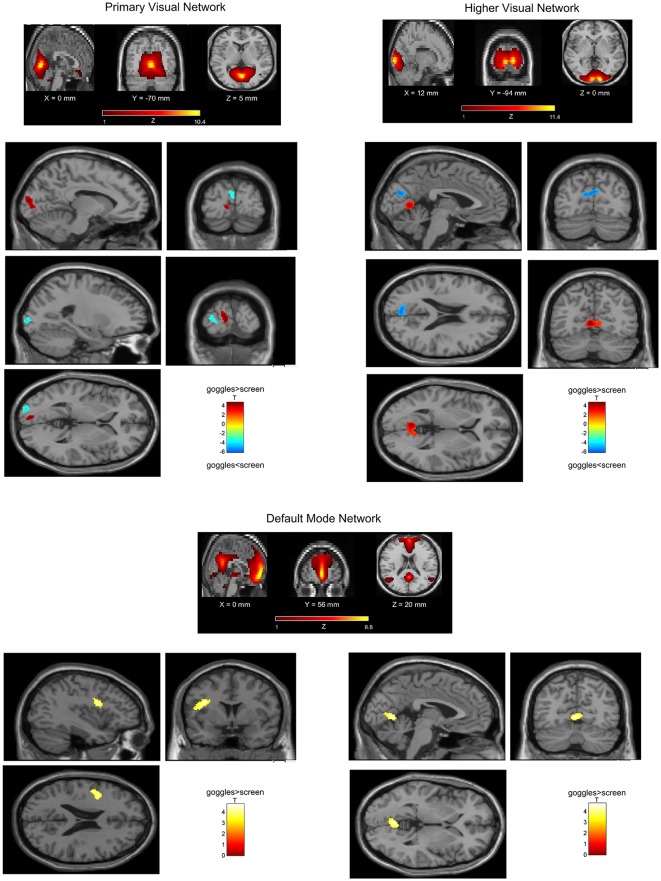
Network spatial maps and group differences: default mode network (DMN) and visual. In the primary visual network, there was an increase in connectivity (in red) in the left calcarine and in the higher visual network in the left lingual in the goggles (monoscopic + stereoscopic) condition as compared to the screen condition. A decreased in connectivity (in blue) was seen in the left middle occipital in the primary visual network and in the bilateral cuneus in the higher visual network. In the DMN, we found an increase (in yellow) in the connectivity in the inferior frontal and bilateral lingual for goggles (monoscopic + stereoscopic) as compared to screen condition.

In the visual networks (Figure 3), we found increased connectivity in the primary and higher networks in the left calcarine (−8, −94, 10) and left lingual (−4, −64, 4), respectively, in the goggles (monoscopic + stereoscopic) as compared to screen condition, that is, when using goggles, the grouping of voxels in left calcarine and left lingual were more strongly correlated to the signal of the source identified, respectively, as primary and higher visual network. A decrease in connectivity was observed in the left middle occipital (6, −90, 30) in the primary visual network and in the bilateral cuneus (0, −78, 24) in the higher visual one, which means, when using goggles, the grouping of voxels in left middle occipital and in the bilateral cuneus were less correlated to the signal of the sources identified, respectively, as primary visual and higher visual network. Investigating the DMN (Figure 3), we found an increase in the connectivity in the inferior frontal (−34, 0, 28) and bilateral lingual (−2, −66, 0) for goggles (monoscopic + stereoscopic) as compared to screen condition, meaning stronger correlation of these areas and the signal of the source identified as DMN. No significant difference between the contrast monoscopic vs. stereoscopic was found in the visual networks nor in the DMN. FDR was used to account for multiple comparison correction due to multiple networks.

### SeedFC

**Figure 4:**
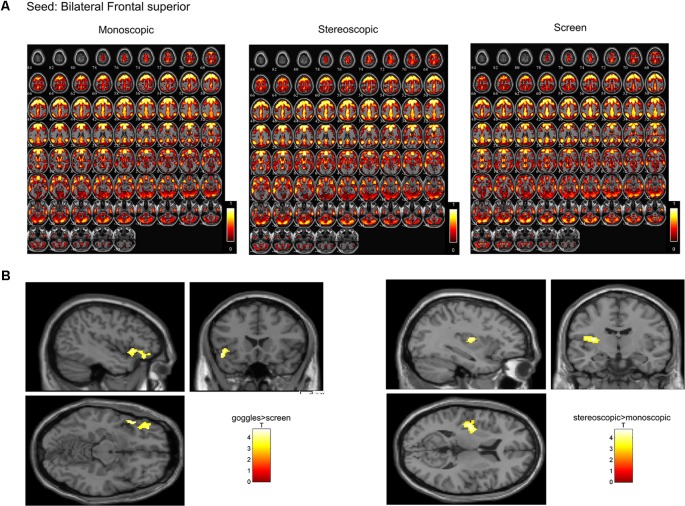
**(A)** Mean seed-based functional connectivity (SeedFC) maps per condition, seed located in the bilateral frontal superior cortex. **(B)** Left—Group differences in SeedFC in goggles vs. screen condition. There was stronger connectivity between a seed in the bilateral superior frontal cortex and the left superior temporal lobe for the magnetic resonance imaging (MRI) goggle contrast goggles (monoscopic + stereoscopic) as compared to the screen. **(B)** Right—Group differences in stereoscopic vs. monoscopic condition. The stereoscopic view elicited stronger connectivity between the bilateral frontal cortex and left insula and putamen.

**Figure 5:**
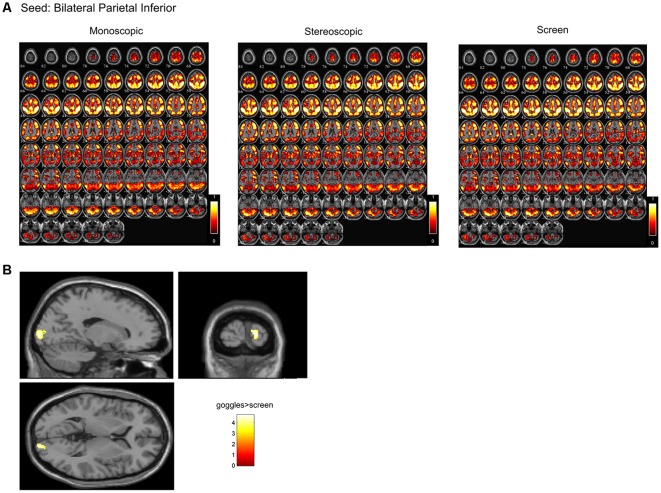
**(A)** Mean seed-based functional connectivity (SeedFC) maps per condition, seed located in the bilateral inferior parietal cortex. **(B)** Group differences in SeedFC in goggles vs. screen condition. There was stronger connectivity between a seed in the bilateral parietal inferior cortex to right calcarine cortex and to right lingual cortex for the MRI goggle contrast goggles (monoscopic + stereoscopic) as compared to the screen.

In a ROI-based seed analysis, we used the following ROIs: bilateral superior frontal cortex, middle frontal cortex, hippocampus, superior parietal cortex, inferior parietal cortex. We found significant seed-based connectivity (Figure 4) between bilateral superior frontal cortex and the left superior temporal lobe (−48, 16, −12) for the MRI goggle contrast goggles (monoscopic + stereoscopic) > screen and to left insula and putamen (−34, 10, 10) in the stereoscopic view contrast (stereoscopic > monoscopic; Figure 4). The bilateral inferior parietal cortex was more strongly connected to right calcarine cortex (18, −98, 4; 20, −88, 2) and right lingual cortex (26, −88, −6) in the goggles vs. screen condition (Figure 5). All seed-based ROI analysis results were FWE corrected at *p* < 0.05. To account for multiple comparison correction due to multiple seeds, FDR was used.

### Graph Analysis

**Figure 6:**
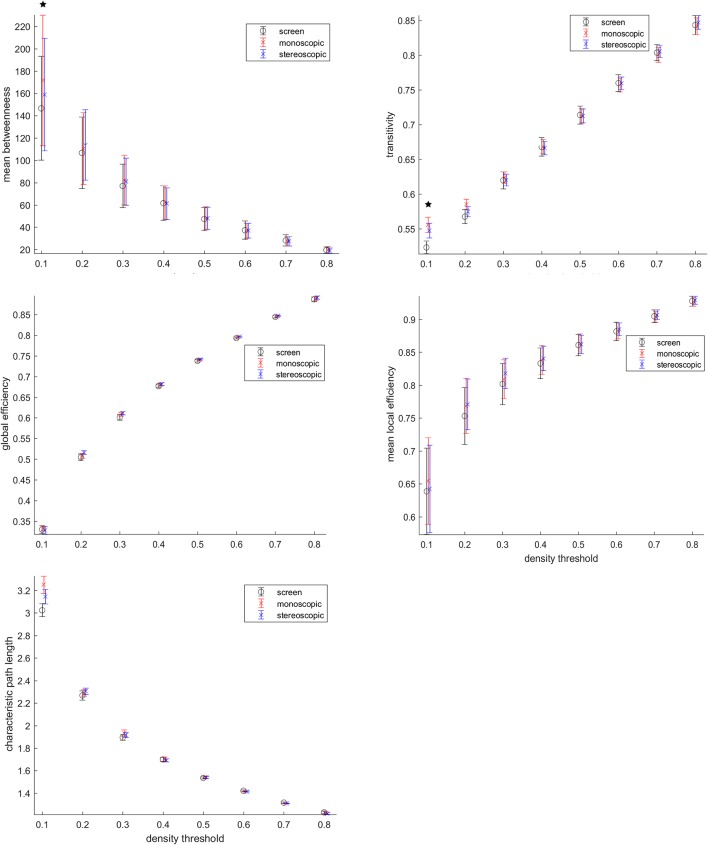
Graph analysis. Betweenneess, transitivity, global efficiency, local efficiency and characteristic path length were calculated. *Means group differences before multiple comparison correction (*p* < 0.05 uncorrected). Group differences were not significant after multiple comparison correction.

Despite no topological differences consistent across threshold were found (Figure 6), the threshold 0.1 representing 10% of the highest connections, goggles condition presented higher mean betweenness than screen, as well as transitivity and characteristic path length. Betweenness is a measure, of centrality, transitivity of segregation and characteristic path length a measure of integration. This means that for networks formed by the highest connectivity links during VR, local information processing (transitivity) was higher, whereas, at the same time, the global exchange of information (characteristic path length) in the brain was also increased. In addition, a higher mean betweenness revealed more central nodes in the network. The local efficiency of the network was also higher during VR as compared to screen, when considering the 30% highest connectivity values as nodes. These results reported above were not corrected for multiple comparisons. No significant differences were found after correcting for multiple comparisons.

## Discussion

Within the scope of the present study, we set out to unravel the effects of VR stimulation presented *via* goggles on functional brain connectivity in MRI. In order to do so we used a virtual game in which the player had the task to fly through a natural scene with the goal to make the landscape blossom, which was designed with the goal to decrease negative affect and induce the experience of flow in its players. To disentangle the effects of presenting the visual stimuli *via* MRI-compatible goggles, we compared the goggle stereoscopic and monoscopic condition with the screen condition. We considered that the goggles, which are mounted on the user’s head and have the ability to display stereoscopic images, are objectively more immersive than a back-projection of a Screen *via* a mirror system (Slater and Wilbur, 1997) and therefore can elicit the sense of presence (Gaebler et al., 2014). As in the back-projection of a screen, the participant receives only 2D images and can still see the scanner bore and oftentimes even the staff operating the scanner next to the presented stimuli. In order to investigate differences in functional brain connectivity during gaming induced by the stereoscopic view, namely the fact that the image is rendered separately for each eye creating the illusion of depth and 3D effect, we contrasted the stereoscopic and monoscopic condition directly. With the goal to obtain a comprehensive picture of brain connectivity we chose four common approaches to analyze resting state fMRI data, namely the assessment of the amplitude of low frequency fluctuation (fALFF; Zou et al., 2008), ICA (Calhoun et al., 2001), seed-based FC analysis and graph analysis (Bullmore and Sporns, 2009).

In line with our hypothesis, the goggles and the stereoscopic contrast revealed stronger brain connectivity for the respective condition with more immersion in the technical (Slater and Wilbur, 1997) and subjective (Gaebler et al., 2014) point of view, that is goggles (stereoscopic and monoscopic) compared to screen and stereoscopic compared to monoscopic generally elicited higher brain connectivity. We found higher fALFF in left cerebellum and postcentral gyrus for goggles compared to the screen. In the visual networks, we found an increase in connectivity in the left calcarine and left lingual for the same contrast, meaning higher correlation of the grouping of voxels in these areas to the signal associated with visual networks and in the DMN there was increased connectivity in the inferior frontal cortex and bilateral lingual gyrus, which means lower correlation of these areas and the signal associated with the DMN activity. Additionally, in the seed-based analysis, we found higher connectivity between bilateral superior frontal cortex and the temporal lobe, as well as bilateral inferior parietal cortex with right calcarine and right lingual cortex for the two goggle vs. screen conditions. Furthermore, we found superior frontal cortex and insula/putamen to be more strongly connected in stereoscopic vs. monoscopic view. When looking at the screen condition, we found higher brain activity, that is higher fALFF values, in right superior orbital frontal cortex.

These results can be viewed as in line with the hypothesis proposed by Jäncke et al. (2009) stating that PFC is involved in the experience of presence. In particular bilateral dorsolateral PFC (DLPFC) activity was shown to be negatively correlated with the subjective report of the experience of presence (Baumgartner et al., 2008) in adults. Moreover, when focusing on connectivity between brain areas, the authors report the results of an effective connectivity analysis from which they conclude that the right DLPFC is involved in down-regulating the activation in the dorsal visual processing stream. Furthermore, the authors interpret the observed increase of activity in the dorsal visual stream during present experience as a sign of higher action preparation in the VR because the brain responds to it similarly as in real-life situations (Jäncke et al., 2009). However, on the same dataset, the left DLPFC was shown to be positively connected to brain regions that are part of the default-mode network (such as medial PFC, anterior cingulate cortex, thalamus, brain stem, nucleus caudatus and parahippocampus). Due to the involvement of the latter brain regions in self-referential processing the interpretation is that higher left DLPFC activation when participants experience less presence leads to an up-regulation of self-referential processing which reflects the detachment from the VR experience.

In contrast to previous studies, in which the focus was on the subjective feeling of presence and brain activity, we set out to investigate differences in brain connectivity between objectively different conditions of stimulation during gaming. A major disadvantage of the previous design, with exception of one (Gaebler et al., 2014), was that the stimulation used to elicit different degrees of presence was not the same. In addition, in all of them, the participants were only passively watching the displays. For this reason, we confronted participants with the same interactive VR game in all three conditions with the only difference being the hardware presentation procedures used to display the respective environment.

Our results can be viewed as being in line with the findings and interpretations shown in association to perceived experience of presence (Baumgartner et al., 2008; Jäncke et al., 2009), since we also find more fALFF in right superior orbital frontal cortex in the 2D screen condition compared to the two goggle conditions. Next to these previous fMRI results our results can also be perceived as in line with results from an EEG study in which the same spatial navigation task in a virtual maze was compared between a projections onto a large wall which was supposedly more immersive than a display on a small Desktop PC screen (Kober et al., 2012). The authors report stronger FC between frontal and parietal brain regions in the Desktop display condition. When it comes to comparing only stereoscopic and monoscopic condition, our results differ from Gaebler et al. (2014) in terms of brain areas. This fact cannot be seen as a surprise considering that they investigated areas that showed common brain activity across subjects by means of the intersubject correlations, which were the temporal lobe, right inferior occipital cortex and right precuneus, whereas we focused on the connectivity between brain areas. Interestingly, the direction of the contrast where differences were found was the same in Gaebler et al. (2014) and in the present study: stereoscopic > monoscopic, meaning that stereoscopic elicited higher intersubject correlations as well as higher connectivity as compared to monoscopic.

However, the rationale for the present study was slightly different from the studies presented before. We set out to test whether overall the stereoscopic VR presentation elicits higher degrees of functional brain connectivity than monoscopic and a screen display.

Our hypothesis was that the condition that elicits the most brain connectivity should be most suited for long-term brain training interventions, assuming that extended training under these conditions could permanently improve brain connectivity on a functional as well as a structural level. Our results show that the majority of contrasts and FC indicators resulting from different analysis pipelines reveal higher brain connectivity between brain regions in the goggle condition and the stereoscopic condition in particular, which we interpret as a hint that training in VR environments in contrast to environments displayed on a screen may be superior in eliciting and therewith facilitating brain connectivity in intervention studies.

At present, a major drawback of the implementation of VR in an MRI environment is the fact that the VR experience is limited to the stereoscopic input to the eyes without the experience of movement in space. Usually, the stereoscopic view in VR is accompanied by the fact that individuals can freely turn their head and move in space while the visual input is adapted to the individual’s movements. However, since the head cannot be freely moved in the MRI scanner, due to its resulting movement artifacts in the images, the actual differences in brain connectivity between a VR and a screen presentation of an environment might be underestimated. Future research may attempt to use motion tracking systems to enable this movement related visual feedback, while at the same time correct for the occurring motion artifacts in the acquired MRI images (Stucht et al., 2015). The limited field of view and resolution of the MRI compatible goggles introduces yet another limitation to such studies. As it can affect the level of immersion for both monoscopic and stereoscopic conditions. Specifically, to the design of the present study, the question still remains of whether and how frequently the participants noticed the 3D effect when, for example, the bee was not flying close enough to the virtual objects.

## Data Availability Statement

The datasets for this manuscript are not publicly available because: it was not part of our ethics statement and therefore the participants were not informed on the fact that the data would be made public. However, the data can be obtained upon request. Requests to access the datasets should be directed to Prof. Dr. SK, s.kuehn@uke.de. The data analysis pipelines are fully available upon request.

## Ethics Statement

The studies involving human participants were reviewed and approved by the local psychological ethics committee of the University Medical Center Hamburg-Eppendorf, Germany. The patients/participants provided their written informed consent to participate in this study.

## Author Contributions

LB, FM, and FS: game development. JG and SK: experimental conception and design. CF and LB: data acquisition. CF and SK: data analysis. CF, LB, FM, FS, JG, and SK: manuscript writing and revision.

## Footnotes

^1^http://thatgamecompany.com/flower/

^2^http://www.mrivideo.com/visuastimdigital.php

^3^http://icatb.sourceforge.net/

^4^https://www.mathworks.com/

^5^http://www.fil.ion.ucl.ac.uk/spm

^6^http://www.fil.ion.ucl.ac.uk/spm
